# Antiviral potential of 3′-sialyllactose- and 6′-sialyllactose-conjugated dendritic polymers against human and avian influenza viruses

**DOI:** 10.1038/s41598-020-57608-4

**Published:** 2020-01-21

**Authors:** Sira Carolin Günther, Julian David Maier, Janine Vetter, Nikita Podvalnyy, Nikolay Khanzhin, Thierry Hennet, Silke Stertz

**Affiliations:** 1 Institute of Medical Virology, University of Zurich, 8057 Zurich, Switzerland; 2 Glycom A/S, Kogle Alle 4, 2970 Hørsholm, Denmark; 3 Institute of Physiology, University of Zurich, 8057 Zurich, Switzerland

**Keywords:** Antivirals, Influenza virus

## Abstract

Current treatment options for influenza virus infections in humans are limited and therefore the development of novel antivirals is of high priority. Inhibiting influenza virus attachment to host cells would provide an early and efficient block of the infection and thus, receptor analogs have been considered as options for antiviral treatment. Here, we describe the rapid and efficient synthesis of PAMAM dendrimers conjugated with either 3′-sialyllactose (3SL) or 6′-sialyllactose (6SL) and their potential to inhibit a diverse range of human and avian influenza virus strains. We show in a hemagglutination inhibition (HAI) assay that human IAV strains can be inhibited by (6SL)- and to a lesser extent also by (3SL)-conjugated PAMAM dendrimers. In contrast, avian strains could only be inhibited by (3SL)-conjugated dendrimers. Importantly, the differential sensitivities of human and avian IAV to the two types of sialyllactose-conjugated dendrimers could be confirmed in cell-based neutralization assays. Based on our findings, we suggest to further develop both, (3SL)- and (6SL)-conjugated PAMAM dendrimers, as influenza virus inhibitors.

## Introduction

Influenza A virus (IAV) is the causative agent of influenza, a respiratory febrile disease in humans that is of medical and economic importance^1^. Vaccines are available to prevent influenza but unfortunately, the current vaccines come with severe limitations, such as reduced efficacy in the elderly or narrow specificity^2^. Hence, there is still a high demand for antivirals against influenza. For many years, two classes of antivirals were approved for clinical use: inhibitors of the viral ion channel M2 and inhibitors of the viral neuraminidase (NA). However, all strains of IAV currently circulating in humans are resistant to the M2 inhibitors, which leaves us with the NA inhibitors^3,4^. Unfortunately, the past has shown that widespread resistance against NA inhibitors can occur with minimal fitness costs^5–7^. Recently, a new drug for the treatment of influenza has been approved in Japan and the U.S.^8^ but rapid selection for drug resistant virus variants has been described for these endonuclease inhibitors^9,10^. Therefore, it is of high priority to develop novel antiviral agents. One promising strategy is to interfere with receptor binding of IAV as such treatment could block the virus early and thereby efficiently^11^. IAV binds to cellular glycoproteins and glycolipids carrying terminal sialic acid (SA) residues via its envelope protein hemagglutinin (HA)^12^. Human isolates of IAV display a preference for SA in an α2,6-linkage to galactose, the penultimate sugar moiety, whereas avian IAV preferentially bind α2,3-linked SA^13^. Numerous SA-based inhibitors have been evaluated for their potential to inhibit IAV (reviewed in^14^). Early on, it became clear that unconjugated monomeric sialosides bind only weakly to HA and display low stability resulting in the need for non-physiological, potentially toxic concentrations to efficiently inhibit virus infection^15–17^. Conjugation of SA to polymers has therefore been explored and proven more promising as higher affinity binding and improved stability can be achieved^17–22^. Dendritic polymers, such as polyamidoamine (PAMAM) dendrimers, can be conjugated to diverse ligands and represent one option to display SA in a multivalent manner^23^.

The initial studies showed that hemagglutination inhibition of IAV could be achieved with SA-conjugated PAMAM dendrimers and that higher valency resulted in better inhibition^17,22^. Kwon and colleagues developed this approach further by coupling 6′-sialyllactose (6SL), a representative of the human-type IAV receptor, to PAMAM dendrimers rather than coupling SA directly to dendrimers^21^. This strategy revealed that not only valency, but also spacing between ligands affects the inhibitory potential. Interestingly, it was observed that different human strains of IAV displayed different sensitivities to inhibition by SA-PAMAMs^22^ but the number of strains tested was limited and the reasons behind differential sensitivity were unclear. Furthermore, only (6SL)-PAMAMs but not 3′-sialyllactose (3SL)-conjugates, which would represent the avian-type receptor specificity, have been tested so far.

Here, we synthesized PAMAM dendrimers branched with either 3SL or 6SL using a rapid and efficient method and assessed their potential to inhibit a diverse range of human and avian influenza virus strains. We show that hemagglutination of human IAV strains can be inhibited by (6SL)- but also (3SL)-conjugated PAMAM dendrimers, whereas avian strains were only sensitive to (3SL)-PAMAMs. Importantly, these differential sensitivities of avian- and human-derived strains of IAV could be confirmed in cell culture-based neutralization assays. In summary, we conclude that both, (3SL)- and (6SL)-conjugated PAMAM dendrimers, should be further developed as IAV inhibitors.

## Results

### Sialyllactose-conjugated PAMAM dendrimers inhibit influenza virus hemagglutination

**Figure 1:**
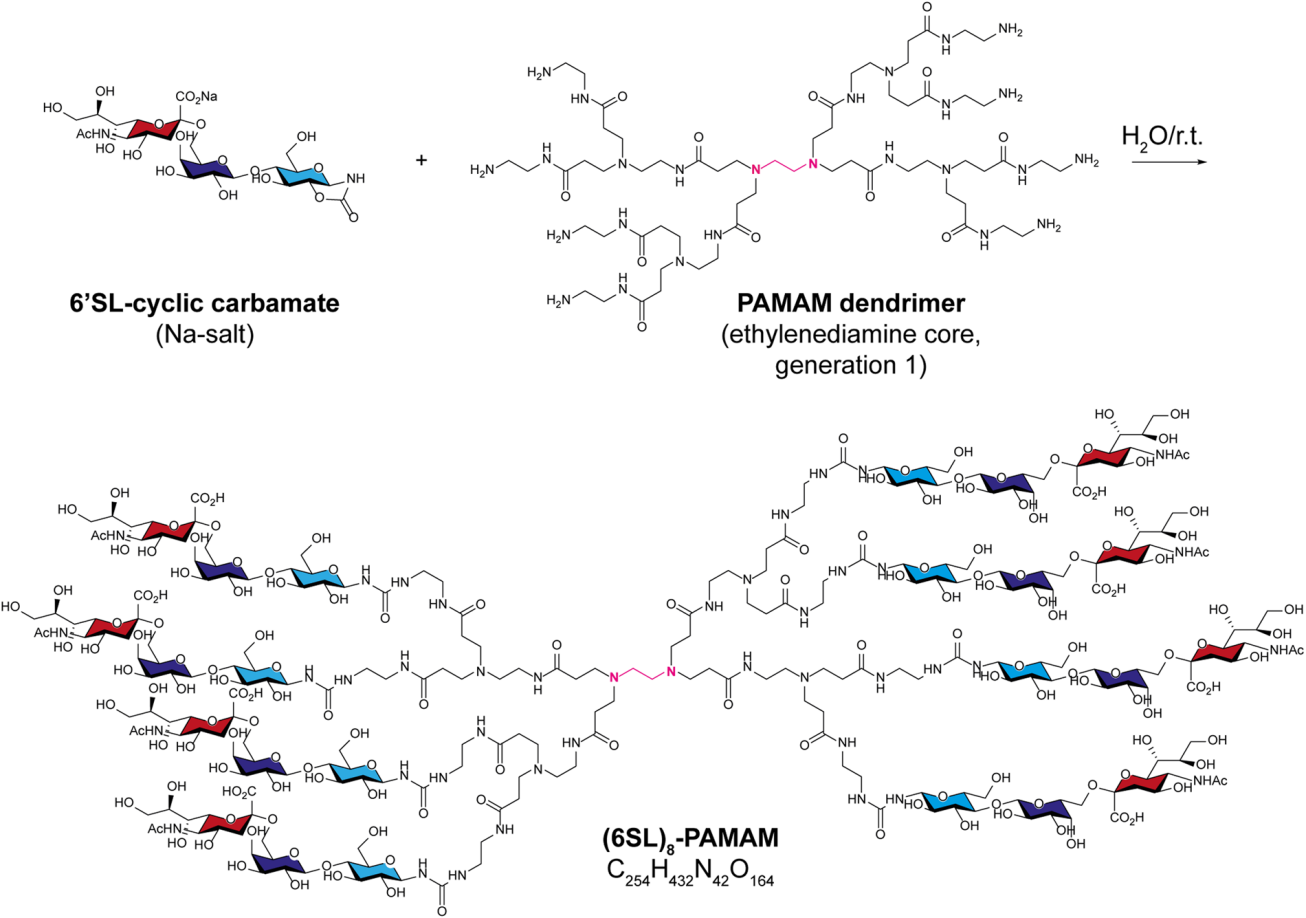
Synthesis of the (6SL)_8_-PAMAM dendrimer. A commercial PAMAM dendrimer with ethylenediamine core depicted in pink and eight primary amino groups (generation 1 (G1)) was coupled to 6′-sialyllactose (6SL)-cyclic carbamate derivatives (sialic acid is colored in red and lactose in blue with galactose in dark and glucose in light blue) in water at ambient temperature in 7 hours. The other three conjugates discussed in this article ((3SL)_4_-, (3SL)_8_- and (6SL)_4_-PAMAMs) were prepared analogously from tetravalent or octavalent PAMAMs with ethylenediamine core and corresponding (3SL)- or (6SL)-cyclic carbamates. r.t. = room temperature.

**Figure 2:**
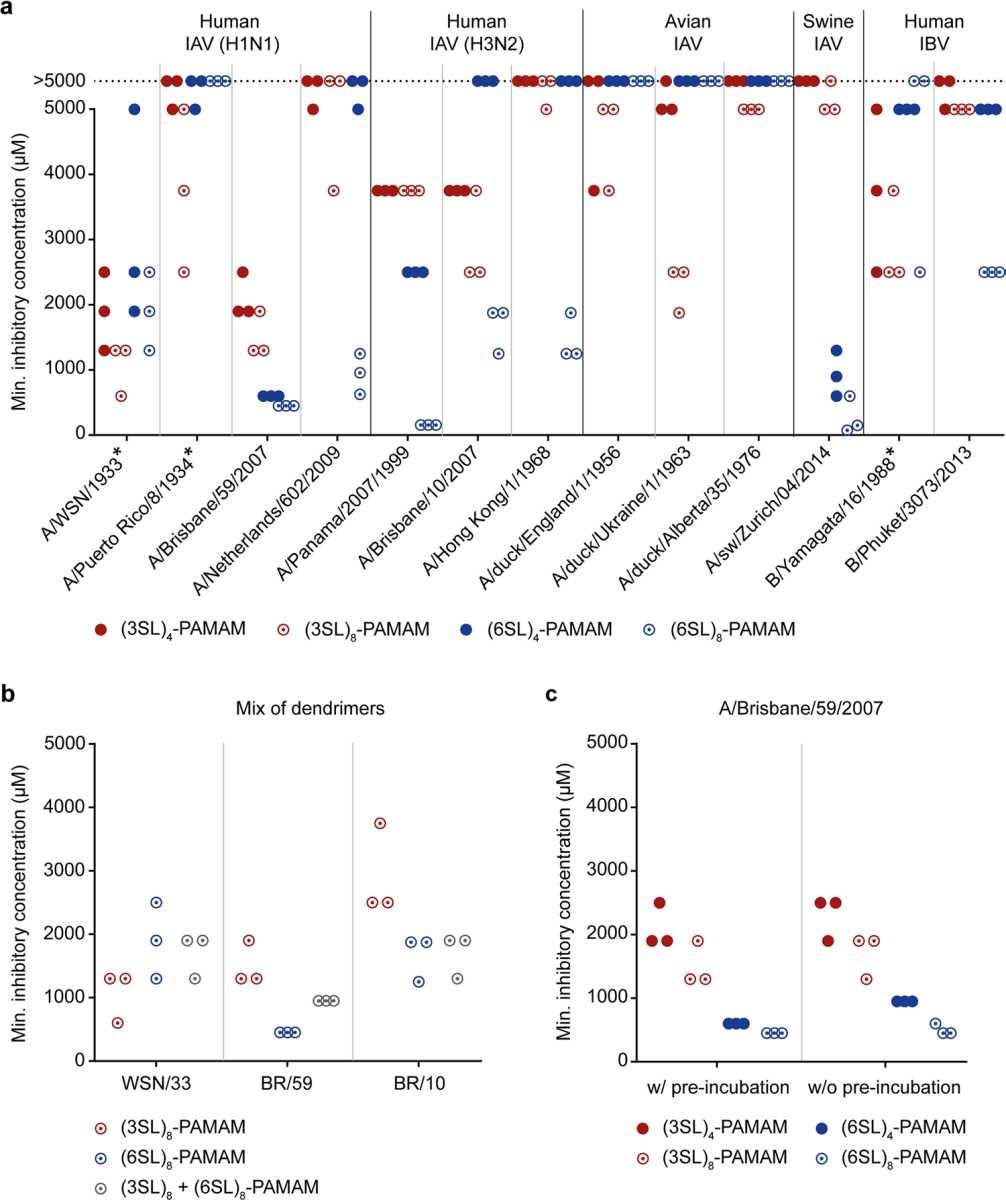
Sialyllactose-conjugated dendrimers possess hemagglutination inhibition activity. **(a)** The minimal inhibitory concentration of PAMAM dendrimers conjugated to either 3′-sialyllactose (3SL) or 6′-sialyllactose (6SL) with either four or eight surface groups ((3SL)_4_-, (3SL)_8_-, (6SL)_4_- and (6SL)_8_-PAMAMs) against indicated viruses (asterisks indicate lab-adapted strains) was determined by hemagglutination inhibition (HAI) assay. If no inhibition was observed at concentrations of ≤5000 µM, a data point was placed on a dotted line indicating a minimal inhibitory concentration of >5000 µM. **(b)** The minimal inhibitory concentration of the two octavalent PAMAM dendrimers ((3SL)_8_- and (6SL)_8_-PAMAMs) against (A/WSN/1933 (WSN/33), A/Brisbane/59/2007 (BR/59) and A/Brisbane/10/2007 (BR/10)) was determined in combination or individually by HAI assay. **(c)** The minimal inhibitory concentration of (3SL)_4_-, (3SL)_8_-, (6SL)_4_- and (6SL)_8_-PAMAMs was determined by HAI assay either with (w/) or without (w/o) pre-incubation of virus (A/Brisbane/59/2007) and PAMAM dendrimers. **(a**–**c)** For each condition, the results of three independent experiments are displayed on a scatter plot (one dot represents one experiment resulting in three dots per dendrimer per condition). Lactose-PAMAM lacking sialic acid residues was included as negative control and displayed no HAI activity at any of the concentrations tested.

The trisaccharides 3SL and 6SL were first derivatized at their reducing end to cyclic carbamate^24^, which enabled the efficient conjugation of the sialyllactoses to primary amines in PAMAM backbones (Fig. 1). We then tested the tetrameric and octameric sialyllactose-conjugated dendrimers ((3SL)_4_-, (6SL)_4_-, (3SL)_8_- and (6SL)_8_-PAMAMs) for their ability to prevent influenza viruses from binding to their receptor in a hemagglutination inhibition (HAI) assay (Fig. 2a). Lactose-conjugated PAMAM lacking sialic acid residues served as negative control and did not show HAI activity with any virus at any of the concentrations tested. We first tested the compounds against two lab-adapted (A/WSN/1933 (WSN/33) and A/Puerto Rico/8/1934 (PR/8)) and two seasonal (A/Brisbane/59/2007 (BR/59) and A/Netherlands/602/2009 (NL/09)) human-derived influenza strains of the H1N1 subtype. We observed that, except for PR/8, at least one of the four inhibitors works well in the low millimolar range with the octavalent compounds being more potent than the tetravalent. Interestingly, the lab-adapted H1N1 subtypes displayed better inhibition with (3SL)-conjugated PAMAMs, whereas the seasonal H1N1 strains were more potently inhibited by the (6SL)-linked PAMAMs although all strains are of human origin. Next, we tested human strains of the H3N2 subtype (A/Panama/2007/1999 (PA/99), A/Brisbane/10/2007 (BR/10) and (A/Hong Kong/1/1968 (HK/68)) and detected a more potent antiviral activity for the octavalent dendrimers conjugated to the human-type receptor. Strain PA/99 was particularly sensitive to HAI by (6SL)_8_-PAMAM with a minimal inhibitory concentration of <200 µM. When testing avian strains of influenza viruses (A/duck/England/1/1956 (EN/56), A/duck/Ukraine/1/1963 (UK/63) and A/duck/Alberta/35/1976 (AL/76)), we could not detect any antiviral activity for the (6SL)-conjugated dendrimers. In contrast, the (3SL)-conjugated dendrimers, in particular the octavalent PAMAM, could inhibit all avian strains tested irrespective of the viral subtype. However, the minimal inhibitory concentration for all avian IAV strains was quite high (>2000 µM) compared to the human isolates. In addition to human and avian IAV strains, we also tested one swine IAV (A/sw/Zurich/04/2014 (Zu/14)) that showed a strong preference for (6SL)-conjugated PAMAM dendrimers in the HAI assay. Moreover, we also included a lab-adapted and a seasonal influenza B virus (IBV) strain (B/Yamagata/16/1988 and B/Phuket/3073/2013) and found that the lab-adapted IBV was inhibited by all four PAMAM variants with a slight preference for those conjugated to the avian-type receptor, whereas the seasonal IBV could only be inhibited by (6SL)_8_-PAMAMs.

Next, we chose three human-derived virus strains – one lab-adapted H1N1 (WSN/33), one seasonal H1N1 (BR/59) and one seasonal H3N2 subtype (BR/10) – that were most sensitive in the HAI assay and assessed the antiviral activity of a combination of (3SL)_8_- and (6SL)_8_-PAMAMs (Fig. 2b). A mixture of the two octamers lead to an intermediate degree of inhibition of all virus strains. We also performed the HAI assay with BR/59 and all four compounds with (w/) and without (w/o) pre-incubation of virus and inhibitor and found that the pre-incubation step slightly ameliorates the minimal inhibitory concentration (Fig. 2c).

### Sialyllactose-conjugated PAMAM dendrimers minimally interfere with NA activity

**Figure 3:**
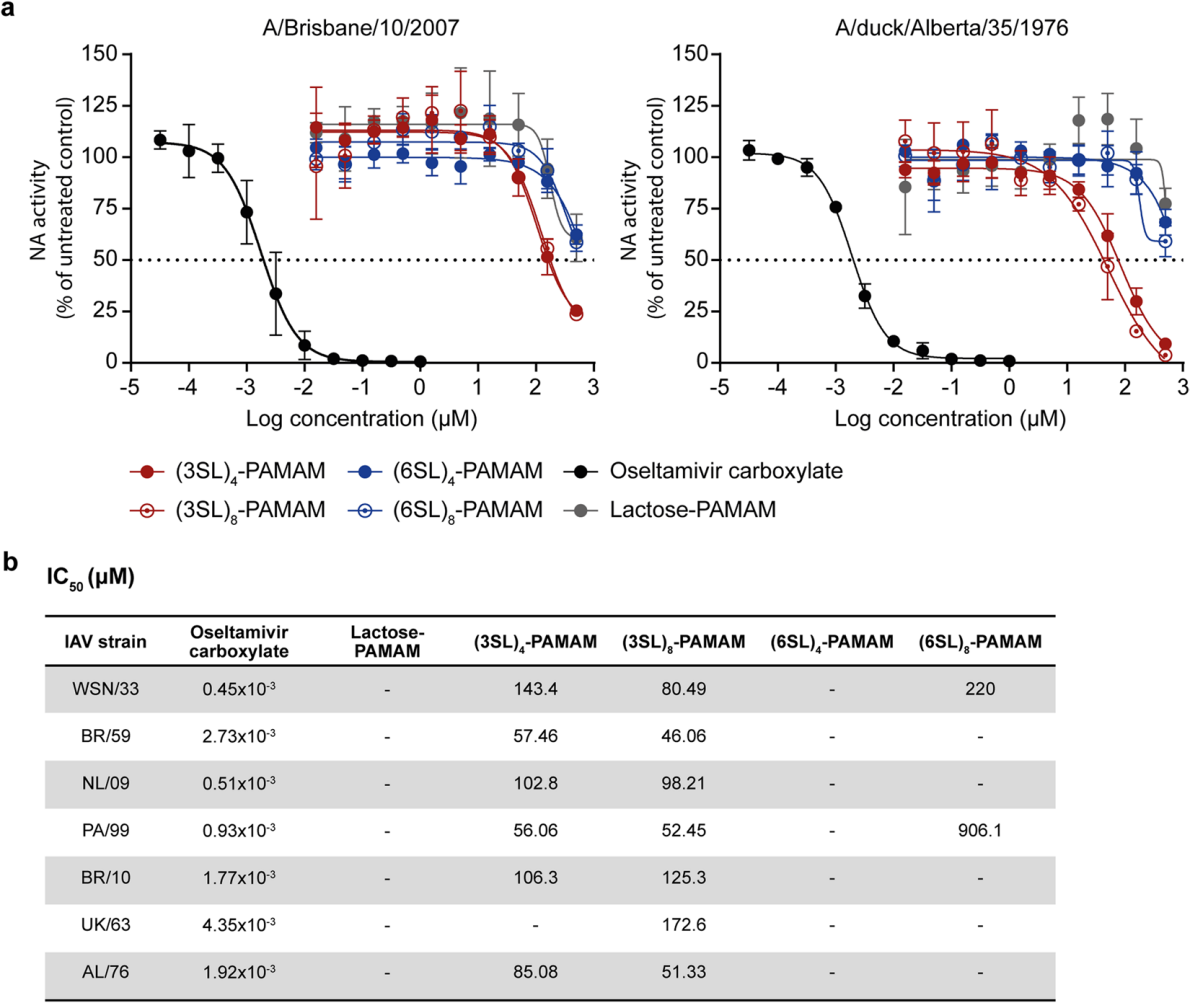
Sialyllactose-conjugated dendrimers minimally interfere with neuraminidase function. (**a)** The neuraminidase (NA) activity of different IAV strains in the presence of PAMAM dendrimers conjugated to either 3′-sialyllactose (3SL) or 6′-sialyllactose (6SL) with either four or eight surface groups ((3SL)_4_-, (3SL)_8_-, (6SL)_4_- and (6SL)_8_-PAMAMs) was measured using the NA-Star Influenza Neuraminidase Inhibitor Resistance Detection Kit. Oseltamivir carboxylate served as positive control and Lactose-PAMAM lacking sialic acid residues was included as negative control. Values were normalized to untreated samples and fitted curves of A/Brisbane/10/2007 and A/duck/Alberta/35/1976 are shown. Error bars indicate the standard deviation (SD) of three independent experiments. **(b)** IC_50_ values in µM for all used IAV strains (A/WSN/1933 (WSN/33), A/Brisbane/59/2007 (BR/59), A/Netherlands/602/2009 (NL/09), A/Panama/2007/1999 (PA/99), A/Brisbane/10/2007 (BR/10), A/duck/Ukraine/1/1963 (UK/63) and A/duck/Alberta/35/1976 (AL/76)).

In a next set of experiments, the inhibitors were analysed for their potential to interfere with NA activity of human IAV strains of the H1N1 subtype (WSN/33, BR/59 and NL/09), the H3N2 subtype (PA/99 and BR/10) and two avian IAV strains (UK/63 and AL/76). Besides all four sialyllactose-conjugated dendrimers ((3SL)_4_-, (3SL)_8_-, (6SL)_4_- and (6SL)_8_-PAMAMs), the Lactose-PAMAM without sialic acid residues on the surface was used as negative control and oseltamivir carboxylate (OC), a NA inhibitor, was used as positive control. As shown in Fig. 3a, NA activity of human BR/10 and avian AL/76 was minimally inhibited by (3SL)- but not (6SL)-conjugated PAMAM dendrimers. The IC_50_ values for all tested viruses and compounds are listed in Fig. 3b. The (3SL)-conjugated PAMAMs displayed low inhibitory activity for all tested viruses with IC_50_ values in the range of 50 to 170 µM for the (3SL)_8_-PAMAM. The (6SL)_8_-PAMAM only reached 50% inhibition for WSN/33 (IC_50_ 220 µM) and PA/99 (IC_50_ 906 µM) but not for any other tested virus.

### Sialyllactose-conjugated PAMAM dendrimers inhibit IAV infection in cell culture

**Figure 4:**
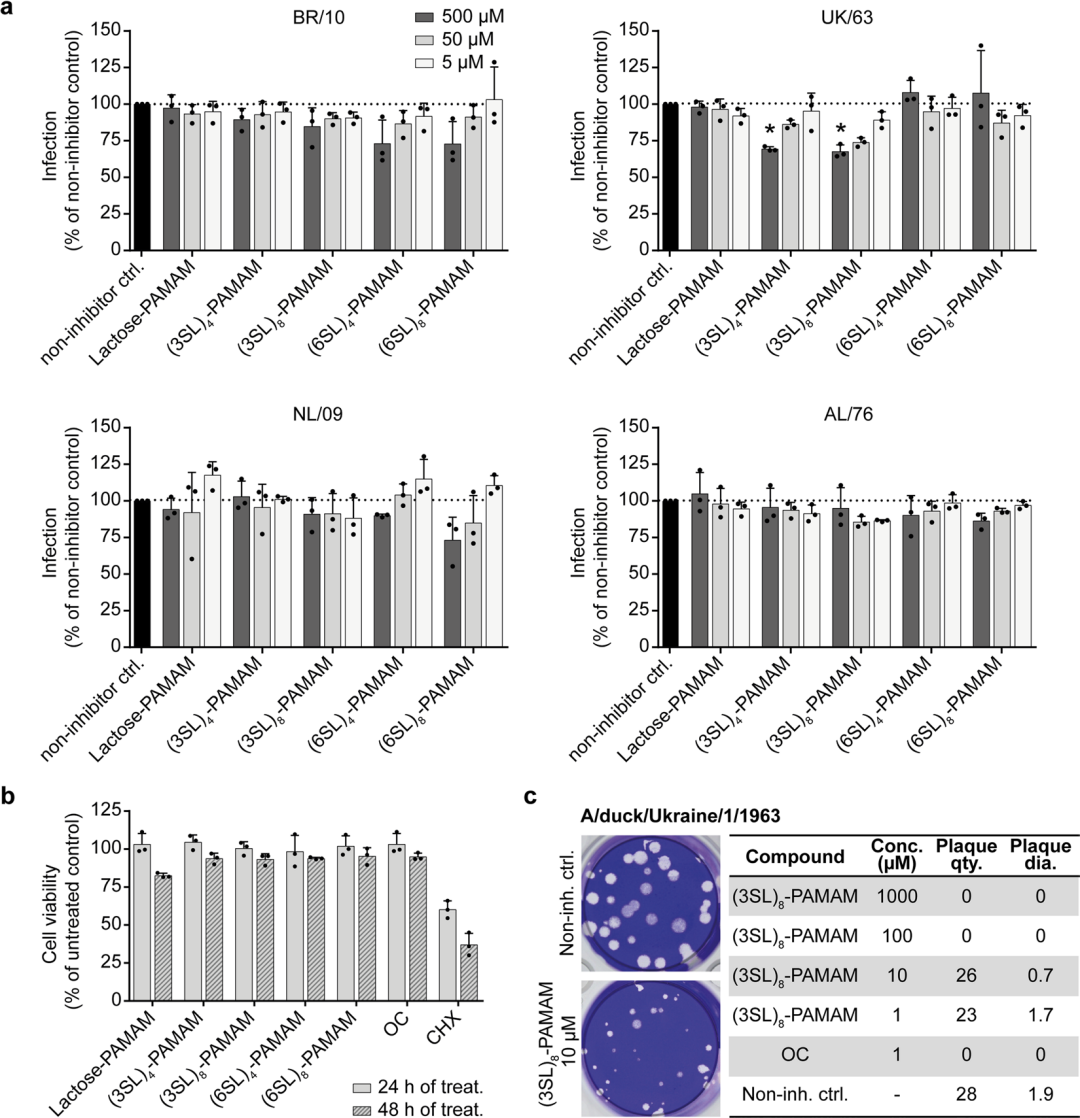
Sialyllactose-conjugated dendrimers inhibit influenza A virus in cell-based assays. (**a**) PAMAM dendrimers conjugated to lactose with no sialic acid residues (Lactose-PAMAM) or to 3′-sialyllactose (3SL) or 6′-sialyllactose (6SL) with either four or eight surface groups ((3SL)_4_-, (3SL)_8_-, (6SL)_4_- and (6SL)_8_-PAMAMs) were pre-incubated with virus (A/Brisbane/10/2007 (BR/10), A/Netherlands/602/2009 (NL/09), A/duck/Ukraine/1/1963 (UK/63) or A/duck/Alberta/35/1976 (AL/76)) for 1 hour at 4 °C. MDCK cells were infected with the compound-virus mix at low multiplicity of infection (MOI) for 1 hour at 37 °C. After removing the inoculum, cells were incubated for up to 24 hours at 37 °C in infection medium containing PAMAM dendrimers. Cells were fixed and stained for the viral nucleoprotein (NP) and DAPI to counterstain the nucleus. The NP signal was quantified and values were normalized to non-treated but infected samples (non-inhibitor control (non-inh. ctrl.)). Error bars indicate the standard deviation (SD) of three independent experiments, each being performed in triplicates. Significance was determined by one-way ANOVA (*P <0.05). **(b)** MDCK cells were treated with 1 mM of the different PAMAM dendrimers (Lactose-, (3SL)_4_-, (3SL)_8_-, (6SL)_4_- and (6SL)_8_-PAMAMs), the negative control oseltamivir carboxylate (OC) or the positive control cycloheximide (CHX), which is known to be cytotoxic, for either 24 or 48 hours. After treatment, cell viability was assessed using the CellTiter-Glo assay. Error bars indicate the standard deviation (SD) of three independent experiments. **(c)** Confluent MDCK cells were infected with 30 plaque-forming units of A/duck/Ukraine/1/1963 for 1 hour at 37 °C before the agar overlay containing the indicated concentrations (conc.) of the positive control OC, the (3SL)_8_-PAMAM or no inhibitor (negative control) was added and incubated at 37 °C until plaques were visible. Cells were then fixed and stained with crystal violet and plaques were counted (plaque quantity (qty.)) and measured (plaque diameter (dia.) in mm). Mean values with standard deviation (SD) of three independent experiments are listed.

Next, we examined the antiviral potential of the PAMAM dendrimers against two human (BR/10 and NL/09) and two avian (UK/63 and AL/76) IAV strains in a cell-based microneutralization (MN) assay (Fig. 4a). The results of the MN assay are in line with those of the HAI assay. We observed inhibition of strain BR/10 by both (6SL)-linked PAMAM dendrimers, whereas strain NL/09 was only inhibited by (6SL)_8_-PAMAM. However, the reductions in infection efficiency did not reach statistical significance. In contrast, avian IAV UK/63 was more sensitive to (3SL)-linked PAMAMs and inhibition was statistically significant for the highest concentration. Cells infected with avian AL/76 did not respond to any of the PAMAM dendrimers, which is also in line with the results of the HAI assay. The inhibitory effects of the PAMAM dendrimers on the different IAV strains are not due to cytotoxicity of the inhibitors as treatment with 1 mM inhibitor for up to 48 hours did not reduce cell viability compared to the negative control, OC, and the positive control, cycloheximide (CHX) (Fig. 4b).

To strengthen the results of the MN assay, we performed an additional cell-based assay, a plaque reduction assay, with UK/63 and the (3SL)_8_-PAMAM as these conditions showed the strongest inhibition of viral infection in the MN assay (Fig. 4c). A complete abrogation of plaque formation was observed for the positive control OC. Strikingly, also the (3SL)_8_-PAMAM caused a reduction in plaque size when used at concentrations of ≥10 µM and completely prevented plaque formation at concentrations of ≥100 µM. These data show that (3SL)- and (6SL)-conjugated PAMAM dendrimers also show antiviral activity in cell-based assays.

## Discussion

In this study, we synthesized (3SL)- and (6SL)-conjugated PAMAM dendrimers and tested them for activity against a broad range of influenza viruses. The HAI assay demonstrated that influenza viruses of human or swine origin were inhibited in binding to their receptors exposed on chicken erythrocytes by (6SL)-conjugated PAMAM dendrimers and to a lesser extent by (3SL)-conjugated PAMAM dendrimers, whereas avian IAV strains were only inhibited by dendrimers carrying the avian-type receptor. Nevertheless, strains WSN/33, PR/8 and B/Yamagata/16/1988 form an exception as they are of human origin but displayed higher sensitivity to (3SL)-PAMAMs. This can be explained by the long history of egg passaging of these three lab-adapted viruses, which resulted in avian-type receptor specificity^13,25^. Furthermore, the data of the human H3N2 viruses also showed that sensitivity to the PAMAM-based inhibitors correlates with binding affinities of the viruses to their receptor: strain PA/99, shown to display much stronger glycan binding than strains HK/68 and BR/10, was most sensitive to sialyllactose-conjugated dendrimers^26^. The sialyllactose-conjugated PAMAM dendrimers could thus be used to determine receptor specificity and potentially also receptor affinity of influenza viruses.

As for some human-derived influenza viruses both the (3SL)- and the (6SL)-conjugated PAMAM dendrimers showed an inhibitory effect, we repeated the HAI assay for one of those viruses (BR/59) by mixing the (3SL)_8_- and the (6SL)_8_-PAMAMs but unfortunately, the combination did not result in a reduction of the minimal inhibitory concentration. Hence, a PAMAM dendrimer conjugated to both α2,3- and α2,6-linked SA might not increase the inhibitory effect but such broad-specificity inhibitors might be of advantage to inhibit strains of unknown tropism and those with dual receptor specificity, like the avian influenza A(H5N1)^27^ or A(H7N9)^28^ virus.

We also performed a NA activity assay to investigate whether the inhibitors are able to interfere with NA activity. Given that the dendrimers present sialic acid in its natural oligosaccharide context, we did not expect to observe strong inhibition and indeed, the sialyllactose-conjugated PAMAM dendrimers only slightly affected NA activity of the different IAV strains. Even though inhibition was minimal, it was interesting to see that the (3SL)-conjugated polymers were more potent than the (6SL)-conjugated polymers against both, avian- and human-derived IAV strains. This is in line with published observations for N2 and N1 NA proteins of human viruses (H3N2 and H1N1) that prefer cleaving avian- over human-type receptors^29,30^ and indicates that the specificity of the NA protein does not need to fully match that of the corresponding HA protein.

To confirm the results from the *in vitro* assays in cell culture, we performed a MN assay and a plaque reduction assay to examine the potential of the PAMAM dendrimers to inhibit viral infection. In general, the HAI assay seems to be a good predictor for the cell-based MN assay although the effects in cell culture are rather small. Higher concentrations of the inhibitors might have increased the inhibitory effect of the PAMAMs but concentrations above 1 mM are not desirable for *in vivo* applications. Therefore, it would make sense to increase the number of surface groups rather than increasing the concentration as Kwon *et al*. could show that a PAMAM dendrimer of generation 4 (G4) with 64 surface groups and an optimized spacing between the (6SL) ligands showed the strongest binding to HA and the best inhibition of H1N1 infection^21^. The PAMAM dendrimers used in this study (generation 0 (G0) with 4 surface groups and generation 1 (G1) with 8 surface groups) are useful for proof-of-concept tests but those with a higher valency and the optimal interligand spacing should be further developed and envisaged for *in vivo* use.

In general, sialyllactose-conjugated dendrimers represent an attractive option for the development of novel antivirals for influenza as they mainly target HA, thereby enabling their combined application with drugs targeting NA in order to maximize efficiency and reduce the emergence of antiviral drug resistance. However, the potential of PAMAM dendrimers of G0 and G1 is limited due to the high concentrations needed. This limitation can be overcome as shown by Kwon *et al*. who synthesized PAMAM dendrimers of generation 4 and 5 featuring up to 128 (6SL) ligands and observed potent antiviral activity in the low micromolar range^21^. Compared with the very slow incorporation of (6SL) ligands onto PAMAM dendrimers by reductive amination applied by Kwon *et al*., the rapidity and efficiency of cyclic carbamate-mediated ligation employed here will enable the synthesis of large amounts of highly multivalent dendrimers. Overall, our results demonstrate host-specific inhibition of influenza virus infection with (3SL)- and (6SL)-linked PAMAM dendrimers further supporting the strategy to use multivalent inhibitors as treatment and prophylaxis for influenza.

## Materials and Methods

### Inhibitor synthesis

The disaccharide lactose and the trisaccharides 3SL and 6SL were first derivatized at their reducing end to cyclic carbamate^24^, which enabled the efficient conjugation of the saccharides to primary amines in PAMAM backbones. Cyclic carbamate-derivatized lactose, 3SL or 6SL (in excess of 2–3 molar equivalents per amino group of PAMAM) were reacted with commercial PAMAM dendrimers (Sigma-Aldrich; ethylenediamine core, generation 0 or 1) within several hours at ambient temperature in water. The obtained fully derivatized dendrimers (Lactose-PAMAM, (3SL)_4_-PAMAM, (6SL)_4_-PAMAM, (3SL)_8_-PAMAM and (6SL)_8_-PAMAM) were initially purified by ion-exchange chromatography (Lactose-PAMAM on Dowex-50WX4-H^+^ and (6SL)_4_-PAMAM on Dowex-1 × 4-HCO_3_^−^) followed by freeze-drying. However, direct precipitation with methanol from the reaction mixture proved to be the most efficient and simple method of product isolation. Thus, all glycodendrimers were efficiently precipitated by dropwise addition of the reaction mixture to the stirring volume of methanol. The structural integrity of conjugates was confirmed by high-resolution electrospray-ionization mass spectrometry and nuclear magnetic resonance (NMR). Lyophilized PAMAMs were dissolved in Dulbecco’s Modified Eagle Medium (DMEM), filtered through a 0.22 μm filter and stored at −20 °C until use.

### Viruses and cell lines

All virus strains except NL/09 and B/Phuket/3073/2013 were grown in 10-day-old embryonated chicken eggs. The study is exempt from the need for ethical approval under Swiss regulations (Tierschutzverordnung 455.1), as virus growth in the chicken eggs was completed before the end of the second trimester. To confirm that egg-passaging of our virus stocks did not cause changes in receptor specificity, egg-grown virus stocks of BR/10 and BR/59 were selected as representatives and sequenced. Comparison to the respective reference sequence derived from tissue culture grown virus stocks revealed no changes in HA that could affect receptor specificity. Strains B/Phuket/3073/2013 and NL/09 were grown in MDCK cells (The European Collection of Authenticated Cell Cultures (ECACC)). Virus stocks were titered in triplicates by plaque assay on MDCK cells (see below). MDCK cells were grown in DMEM supplemented with 10% fetal calf serum (FCS), 100 U/ml penicillin and 100 µg/ml streptomycin.

### Hemagglutination inhibition (HAI) assay

HAI assays were performed as described previously^31,32^. In brief, two-fold serial dilutions of the inhibitors were pre-incubated in a 96-well plate with 8 HA units of virus per well for 30 min at 4 °C. Chicken erythrocytes (Bell AG), that contain α2,3-linked and α2,6-linked SA receptors on their surface, were added at a final concentration of 0.5%, and the plate was incubated at room temperature (RT) for 45 min. HAI titers were determined as the highest dilution that displayed inhibition of hemagglutinating activity referred to as minimal inhibitory concentration.

### Neuraminidase (NA) activity assay

NA activity was measured using the NA-Star Influenza Neuraminidase Inhibitor Resistance Detection Kit (Applied Biosystems^TM^) according to the manufacturer’s instructions. The values were normalized to the untreated control, which is set to a 100%, and IC_50_ values were calculated by nonlinear regression analysis (GraphPad Prism 7).

### Virus titration by plaque assay

MDCK cells were seeded into 12-well plates. Upon complete confluency, cells were infected with a 10-fold dilution series of virus and incubated for 1 hour at 37 °C with regular shaking of the plates. The agar overlay (25 mL 2x MEM with 9 ml water, 0.5 ml DEAE dextran (1%), 0.75 ml sodium bicarbonate (7.5%), 17.5 ml Oxoid^TM^ purified agar (2%) and 1 µg/ml Tosylamide-2-phenylethyl-chloromethyle-ketone (TPCK)-treated trypsin) was added to each well and cells were incubated at 37 °C until plaques were visible. Cells were fixed with 3.7% paraformaldehyde (PFA) and stained with a crystal violet solution (0.05% crystal violet and 16% methanol in ddH_2_O).

### Plaque reduction assay

Confluent MDCK cells in a 12-well plate were infected with 30 plaque-forming units (PFU) of the indicated virus for 1 hour at 37 °C. After infection, the virus inoculum was removed and replaced by the agar overlay containing the sialyllactose-conjugated dendrimers at different concentrations (1 µM to 1 mM) or OC (1 µM) as positive control. Following incubation at 37 °C until plaques were visible, cells were fixed with 3.7% PFA and stained with crystal violet solution. Finally, plaques were counted and measured.

### Microneutralization (MN) assay

MDCK cells were seeded in a 96-well plate (1.5 × 10^4^ cells/well). The next day, PAMAM dendrimers (5, 50 and 500 µM) were pre-incubated with virus at low multiplicity of infection (MOI) for 1 hour at 4 °C before cells were infected with the compound-virus mix for 1 hour at 37 °C. Following infection, the inoculum was removed and replaced by infection medium (DMEM supplemented with 0.1% FCS, 3% BSA, 20 mM Hepes, 100 U/ml penicillin, 100 μg/ml streptomycin and 1 µg/ml (TPCK)-treated trypsin) containing PAMAM dendrimers at the specific concentrations. After incubation of up to 24 hours at 37 °C depending on the virus kinetics, cells were fixed with 3.7% PFA for 10 min at RT, permeabilized with 0.5% Triton X-100 for 5 min at RT and stained with 4′,6-diamidino-2-phenylindole (DAPI) to visualize the nuclei and a mouse monoclonal antibody against the viral nucleoprotein (HB-65, American Type Culture Collection)^33^. Following incubation with the secondary antibody (goat anti-mouse Alexa Fluor 488; Invitrogen), the NP signal was measured using a microplate reader (Perkin Elmer). The values were normalized to the non-inhibitor control (cells + virus without inhibitor = 100%) and analysed by one-way ANOVA (Graph Pad Prism 7) for comparison of multiple conditions. The P values for significance are stated in the figure legend.

## Data Availability

The authors confirm that the data supporting the findings of this study are available within the paper.
